# P-893. Expedited Transition to Oral Antibiotics in Gram Negative Bacteremia Reduces Hospital Length of Stay

**DOI:** 10.1093/ofid/ofae631.1084

**Published:** 2025-01-29

**Authors:** Zachary Nelson, Alyssa Christensen

**Affiliations:** HealthPartners, Saint Louis Park, Minnesota; HealthPartners, Saint Louis Park, Minnesota

## Abstract

**Background:**

Uncomplicated Gram-negative bloodstream infection (GN-BSI), often originating from the urinary tract, is a common reason for hospitalization in the United States. No high-quality data exist that suggest a specific duration of intravenous treatment is indicated for GN-BSI. As such, we hypothesized that expedited transition to oral antibiotics (at the time of susceptibility testing result availability) may facilitate earlier discharge from the hospital. Efforts to reduce hospital length of stay (LOS) yield benefits to both the patient and the healthcare system and have not been as consistently reported as more traditional stewardship metrics, such as time to optimal treatment. A combination of targeted provider and pharmacist education and formal ASP recommendations documented in the medical record were used to facilitate swift transition to oral antimicrobials.

**Methods:**

Data was collected prior to (January to July 2023) and after (August 2023 to December 2023) the intervention consisting of targeted physician and pharmacist education and formal documentation of ASP recommendations in the medical record was deployed on August 1, 2023. The post-intervention cohort was stratified by whether formal ASP recommendations were documented in the medical record, which may reflect the impact of the targeted education efforts alone. The three groups were compared using an analysis of variance.

**Results:**

The average hospital LOS for the pre-intervention (n = 421), post-intervention without ASP recommendations (n = 260), and post-intervention with ASP recommendations (n = 83) groups were 7.13, 6.84, and 5.84 days, respectively (p = 0.63). While not statistically significant, using a conservative estimate of $1,100 per hospital day yields an estimated cost savings due to reduced LOS alone of approximately $187,200 from August to December 2023 that may be attributable to the aforementioned interventions.

**Conclusion:**

Targeted physician and pharmacist education and documenting formal ASP evidence-based recommendations in the medical record that guide clinicians to transition to oral antibiotics when susceptibility results are available can meaningfully reduce hospital length of stay. The reduction in LOS observed in our study was greatest when the two strategies were combined.

**Disclosures:**

**All Authors**: No reported disclosures

